# Yarning to understand locally tailored dementia prevention and care for Australian Aboriginal and Torres Strait Islander peoples

**DOI:** 10.1002/alz70858_102507

**Published:** 2025-12-25

**Authors:** Antonia Jean Clarke, John Towney, Louise Lavrencic, Cammi Murrup‐Stewart, Kylie Radford, Adrienne Withall, Amy Brodtmann

**Affiliations:** ^1^ Monash University, Melbourne, VIC, Australia; ^2^ University of New South Wales, Sydney, NSW, Australia; ^3^ Neuroscience Research Australia, Sydney, NSW, Australia; ^4^ University of New South Wales, Sydney, Australia; ^5^ Department of Neurology, Royal Melbourne Hospital, Parkville, VIC, Australia

## Abstract

**Background:**

Aboriginal and Torres Strait Islander peoples experience greater dementia prevalence at a younger age than non‐Indigenous Australians. Place influences health outcomes, yet urban‐rural effects on brain ageing remain unclear. Whilst rural regions may lack adequate health resourcing, existing local strengths such as connection to Country, proximity to Community and culturally appropriate care can support healthy brain ageing and dementia care among Aboriginal and Torres Strait Islander peoples living in these areas. This study employed research yarning, an Indigenous methodology, to examine the influence of Place on dementia prevention and care for Aboriginal and Torres Strait Islander peoples.

**Methods:**

Research methodology was designed to ensure cultural safety, integrating Indigenous and Western approaches. Research yarns on dementia prevention and care were held with Aboriginal and Torres Strait Islander and non‐Indigenous community members (*n* = 42) and healthcare professionals (*n* = 24) across one metropolitan, three regional/rural, and two remote sites in New South Wales, Australia. Thematic analysis was conducted by Aboriginal and non‐Indigenous researchers using inductive and deductive coding.

**Results:**

Key themes included Community understanding of brain ageing, relationships between Place and brain ageing, service accessibility, and availability of training relating to dementia care for Aboriginal and Torres Strait Islander peoples. Maintaining cognitive health to share intergenerational experiences is a key foundation of ageing well for Aboriginal and Torres Strait Islander peoples across rural strata. Strengths relating to dementia prevention include the embedding of cardiovascular health in primary care, regular contact with family and Indigenous health providers, and, especially in more rural areas, connection to traditional knowledges. Barriers include limited awareness of brain health strategies among Community and healthcare providers and differing perceptions of declining cognition. Gaps in culturally appropriate approaches impair adequate dementia care with a lack of service availability and coordination more evident in rural areas.

**Conclusions:**

Health for Aboriginal and Torres Strait Islander peoples is holistic and collectivist, reflecting the conditions of Place and Community. With almost half of dementia risk modifiable over the life course, leveraging existing cultural strengths, including the regenerative capacity of Community, especially in rural settings, offers an innovative approach to dementia prevention and care for Aboriginal and Torres Strait Islander peoples.

